# The efficacy of strength or aerobic exercise on quality of life and knee function in patients with knee osteoarthritis. A multi-arm randomized controlled trial with 1-year follow-up

**DOI:** 10.1186/s12891-023-06831-x

**Published:** 2023-09-08

**Authors:** Britt Elin Øiestad, Asbjørn Årøen, Jan Harald Røtterud, Nina Østerås, Even Jarstad, Margreth Grotle, May Arna Risberg

**Affiliations:** 1 Department of Rehabilitation Sciences and Health Technology, Oslo Metropolitan University, Oslo, Norway; 2 Orthopedic department, Akershus University Hospital, Lørenskog, Norway; 3 Orthopaedic department, Akershus University Hospital, Lørenskog, Norway; 4 Center for treatment of Rheumatic and Musculoskeletal Diseases (REMEDY), Diakonhjemmet Hospital, Oslo, Norway; 5 Norwegian Sports Medicine Clinic, Oslo, Norway; 6 Department of Research and Innovation, Division of Clinical Neuroscience, Oslo University Hospital, Oslo, Norway; 7 Division of Orthopedic Surgery, Oslo University Hospital, Oslo, Norway; 8 Department of Sports Medicine, Norwegian of School Sport Sciences, Oslo, Norway

**Keywords:** Knee osteoarthritis, Quality of life, Exercise

## Abstract

**Objective:**

To evaluate the efficacy of strength exercise or aerobic exercise compared to usual care on knee-related quality of life (QoL) and knee function at 4 months and 1 year in individuals with knee osteoarthritis.

**Methods:**

A three-arm randomized controlled trial (RCT) compared 12 weeks of strength exercise or aerobic exercise (stationary cycling) to usual care supervised by physiotherapists in primary care. We recruited 168 participants aged 35–70 years with symptomatic knee osteoarthritis. The primary outcome was The Knee Injury and Osteoarthritis Outcome Score (KOOS) QoL at 1 year. Secondary outcomes were self-reported function, pain, and self-efficacy, muscle strength and maximal oxygen uptake (VO_2max_) at 4 months and 1 year.

**Results:**

There were no differences between strength exercise and usual care on KOOS QoL (6.5, 95% CI -0.9 to 14), or for aerobic exercise and usual care (5.0, 95% CI -2.7 to 12.8), at 1 year. The two exercise groups showed better quadriceps muscle strength, and VO_2max_ at 4 months, compared to usual care.

**Conclusion:**

This trial found no statistically significant effects of two exercise programs compared to usual care on KOOS QoL at 1 year in individuals with symptomatic and radiographic knee osteoarthritis, but an underpowered sample size may explain lack of efficacy between the intervention groups and the usual care group.

**ClinicalTrials.gov Identifier:**

NCT01682980.

**Supplementary Information:**

The online version contains supplementary material available at 10.1186/s12891-023-06831-x.

## Introduction

Knee osteoarthritis is a world-wide leading cause of pain, disability, and reduced quality of life (QoL) [1, 2]. There is no cure for knee osteoarthritis, but exercise interventions have shown beneficial, mostly short-term (post-intervention) effects on pain and function compared to no or minimal treatment, usual care, and placebo treatments [3–9]. Exercises are currently being implemented as the first-line treatment in clinical practice across countries, even though the effect sizes are at best moderate [10, 11]. Exercise interventions involve many different activities, such as strength and balance exercises, walking programs, cycling, or water-based exercises, which make a good base for personalized treatment options. Strength exercises are likely among the most important interventions due to the relatively strong association between lower knee extensor strength and increased risk of symptomatic and functional deterioration in individuals with symptomatic knee osteoarthritis [12]. However, fewer studies have reported the effects of strength training on knee-related QoL in younger populations beyond short-term post-intervention assessments [9]. The knee-related QoL outcome has been proven to be more sensitive and responsive to detect changes in younger and more active populations than pain and physical function [13] and represents a good complement to the pain and function outcomes. Furthermore, the OMERACT-OARSI group suggests that QoL is one of the core outcome domains for measurement in clinical trials of hip and knee osteoarthritis [14].

Also, less is known about the efficacy of aerobic exercises alone on knee-related QoL. Of 48 trials in a systematic review [6], none examined moderate “single-type” exercises such as cycling. Another more recent systematic review [15] summarizing the effects of cycling on pain, function, and stiffness in individuals with knee osteoarthritis concluded that stationary cycling relieves pain and improves sport function, but was not clinically effective for improving stiffness, daily activity, or QoL. The authors requested more trials to clarify the effects of stationary cycling.

There is still lack of evidence on the efficacy of different types of structured, easily available exercise programs beyond post-intervention assessment on knee-related QoL in younger patients with symptomatic knee osteoarthritis. The primary objective of this trial was to evaluate the efficacy of a standardized strength exercise program, or a standardized aerobic exercise program compared to usual care at 1 year on knee-related QoL in individuals with symptomatic knee osteoarthritis. We hypothesized that (1) Strength exercise was more effective than usual care on knee-related QoL at 1 year in individuals with symptomatic knee osteoarthritis, and (2) Aerobic exercise was more effective than usual care on knee-related QoL at 1 year in individuals with symptomatic knee osteoarthritis.

The secondary objectives were to investigate the efficacy of respectively strength exercise or aerobic exercise compared to usual care, on pain, symptoms, function in activities of daily living (ADL) and sport/recreation, self-efficacy for pain and symptoms, health-related QoL, muscle strength and cycle-specific maximal oxygen uptake (VO_2max_) at 4 months and 1 year in individuals with symptomatic knee osteoarthritis.

## Methods

### Trial design

This three-arm parallel randomized controlled trial (RCT) is reported according to the Extension of the CONSORT 2010 Statement [16] and the CHAMP statement [17]. The study was designed to compare each one of the exercise programs to a usual care group on Knee injury and Osteoarthritis Outcome score (KOOS) QoL at 1 year as the primary endpoint [18]. This was described in the clinicaltrials.gov (NCT01682980, 11/09/2012) documents prior to the start of the study. The changes in the trial from the study protocol are [18]: We had to stop the trial before we had included the planned 207 participants because the recruitment rate was slow, and we lacked the resources to conduct the recruitment. With only one person available for the recruitment job throughout the study period, we decided to terminate the trial when Covid-19 restrictions were implemented in Norway. We could not do the planned *magnetic resonance imaging (MRI) assessments and blood samples* of the study participants due to lack of funding to conduct these parts. The inclusion criterion for age was extended from initially 45–65 to 35–70 years of age (after inclusion of 42 participants) to reach out to more eligible and younger participants. We did not initially design the RCT to compare the two exercise interventions as this may have required a huge sample size to detect clinically important differences.

### Participants

Participants with a confirmed diagnosis of symptomatic and radiographic knee osteoarthritis were recruited from primary care, orthopedic departments at three hospitals in the greater Oslo area, and from newspaper advertisements, from April 2013 to March 2020. We recruited participants with confirmed radiographic Kellgren and Lawrence grades [19] 2 and 3 defined using the SynaFlexer frame (Synarc Inc, Newark, CA). The participants had to fulfill 3/4 of the American College of Rheumatology (ACR) clinical criteria (stiffness < 30 min, crepitus, osteophytes, pain the last days of the last month) [20], being aged 35–70 years, and having no other serious physical or mental illnesses preventing them from participating in the trial (e.g. cancer under treatment or unstable coronary heart disease). We excluded eligible participants who self-reported body mass index (BMI) > 35 kg/m^2^ because we believed they needed an additional weight loss program. In addition, we excluded those who were scheduled for surgery in the nearest 6 months, those who already participated in structured, weekly, moderate strength training or cycling, those who had known serious musculoskeletal impairments in the lower extremities or low back, having prostheses in the lower extremities, those with serious coronary heart diseases or cancer, and those who did not speak Norwegian language.

### Randomization and blinding

Computer-generated randomization lists were prepared by a biostatistician not involved in the project. Participants were randomly allocated with 1:1:1 ratio within block sizes of 6. A research coordinator prepared concealed envelopes from the randomization list for four recruitment centers before recruitment (three orthopedic departments and one for primary care involving physical therapy clinics/advertisements). The self-reported data and objective outcomes assessors were blinded for group assignment.

### Assessments and outcomes

A sheet was completed by an assessor and the participant at the time of *recruitment* including data on: age, sex, self-reported height and weight, affected knee (right/left/both), year of diagnosis, osteoarthritis in the family, ACR criteria, knee pain most days the last month (yes/no), scheduled surgery in any joint (yes/no), known severe physical or psychological disorders, drug abuse, physical activity level and physical activity level index (type*frequency* intensity) [21], smoking (yes/no), previous injuries or surgeries in the knees or hips (type of injury/no injury), and known heart diseases for the participant and their parents or siblings (yes/no). Additional data was collected at the *baseline* test: objectively measured height and weight, educational level, work status, and a numeric rating scale of average pain (NRS) in the affected knee last week (0–10). We also assessed frontal plane alignment using an inclinometer [22] and knee range of motion by a goniometer (data not shown).

Assessments were performed before random group allocation (baseline), and at post-intervention (4 months), and at 1 year. The participants started with a warm-up on a stationary cycle. Then the VO_2max_ test was conducted before the isokinetic muscle strength tests. All participants had a 5-10-minute break between the cycle and muscle strength tests. In the end, the participant completed the patient-reported outcomes. This procedure was used for all the tests to ensure consistent order of the physiological tests. We also phoned the participants at 6 months and 9 months for an interview of health care utilization the last three months, including a question regarding the frequency of physiotherapist consultations.

#### Primary outcome

The primary outcome was knee-related QoL measured by the patient-reported KOOS QoL [23] at 1 year. The KOOS is a valid and reliable self-reported questionnaire with five sub-scores measuring pain, symptoms, activities of daily living, function in sport and recreation (KOOS Sport/recreation), and knee-related QoL for patients with knee injuries and osteoarthritis [13]. The subscales range from 0 to 100 with 0 representing the worst possible score, and 100 best possible score.

#### Secondary outcomes measured at 4 months and 1 year

Secondary outcomes were the KOOS subscales (0-100 for pain, other symptoms, activities of daily living (ADL), Sport/recreation, and QoL (at 4 months). Other secondary outcomes were knee pain the previous week (numeric rating scale (NRS) scale, 0–10), health-related QoL measured by EuroQoL-5 Dimentions-5 Levels (EQ-5D-5 L) [24], the 0-100 scale of patient-reported health status, and the EQ-5D-5 L index. The index was calculated using the UK value set as described by Garrett et al. [25]. Self-efficacy for pain (5–25) and function (6–30) was measured using the Arthritis Self-Efficacy Scale (ASES) [26]. The Global rating of change scale (GRC) 7-point version was completed at both follow-ups [27], asking “*how are your knee complaints now compared to the previous assessment*”. Isokinetic knee extension strength was tested in a dynamometer (Biodex 2000 System: Biodex Medical Systems, Shirley, NY) with the participant in a sitting position with belts ensuring that only the knee joint could move. Concentric knee extension and knee flexion in a range of 0–90 degrees with five repetitions at 60°/sec were tested. The assessor gave verbal feedback to all the patients to encourage maximal effort. Muscle strength was quantified based on peak torque in Newton meters (Nm) and the peak torque per kilogram body weight (Nm/kg). Peak torque was reported as the highest value among the five repetitions. VO_2max_ was assessed using an incremental test procedure on a cycle ergometer (Monark 839E, Sweden), after a 20-minute progressive warm-up [28]. For this purpose, two metabolic analyzers were used; a Sensor Medics VMax29 with a mixing chamber (Vyaire Medical, Höchberg, Germany), or a Vyntus CPX with a breath-by-breath system (Vyaire Medical, Höchberg, Germany), where the same analyzer was used at pre- and post-test for correct comparison. During the test procedure, the workload was increased by 25 Watts every 30 s to a supramaximal workload and expected volitional exhaustion within ~ 4–6 min. The cadence was customized individually and was increased from ~ 50–75 revolutions per minute to ~ 90–110 revolutions per minute at peak workload. The main criterion for achievement of VO_2max_ was the classic leveling off of oxygen uptake (VO_2_), despite increased workload [29]. Secondary criteria used to validate the attainment of VO_2max_ and indicate maximal effort during the incremental test was a plateau in VO_2_, despite increased pulmonary ventilation [30], a peak pulmonary respiratory gas-exchange ratio of > 1.10 [31], a rate of perceived exertion of ≥ 17 on the BORG_6 − 20_ scale [30], and visible exhaustion of the subject [32]. For confirmation of a satisfying test procedure, at least two of the five criteria mentioned above had to be met. VO_2max_, reported in mL·kg^− 1^·min^− 1^ in the present study, was calculated as the mean of the two highest consecutive 30-second VO_2_ measurements [33, 34]. Maximal heart rate (HR_max_) was estimated by peak heart rate achieved during the incremental test + 5 beats·min^− 1^ [35] by wearing a heart rate monitor (Polar S210, Polar Electro Oy, Kempele, Finland). A cardiovascular examination was performed prior to the test to exclude those with severe and unstable coronary heart diseases, and an electrocardiogram assessment was included of all participants > 50 years of age during the incremental test.

### Exercise programs and usual care

The exercise interventions are described according to the Consensus on Exercise Reporting Template (CERT) [36] (Appendix File 1). The intervention period started as soon as possible after the baseline assessment with individual follow-up by a physiotherapist with previous clinical experience with osteoarthritis patients. The physiotherapists were located at clinics near the participants’ homes. The physiotherapists received one oral explanation of the project and the two exercise programs, and received a print of the programs, from our research coordinator before meeting their first participant. The physiotherapists could contact our research coordinator for questions at any time point during the intervention period. The physiotherapist clinics were differently equipped, but all had equipment for leg-press and leg-extension exercises, and all had stationary bicycles. Both interventions started with a two-week preparation phase to adapt to the program. Then both groups were told to exercise 2–3 times per week for 12 weeks (at least 2 sessions per week supervised by the physiotherapist and the third could be a home session) according to the American College of Sports Medicine`s (ACSM) guidelines for exercise in untrained people [37]. The participants were told not to change their usual physical activities during the 12-week intervention, but this was not systematically recorded other than self-reporting of physical activity level. The interventions involved exercises only, but the PTs were *not* instructed to refrain from giving advice on lifestyle changes, including continuing the exercise program post-intervention. The participants completed a training diary including details about type of exercise, frequency, intensity, duration, and pain during and after the exercise session (NRS, 0–10). Adequate adherence to the exercise interventions was defined as completing 80% of the total number of sessions prescribed (2 sessions per week*12 weeks = 24 sessions).

#### Strength exercise program

The strength exercise program was an individual, supervised program based on a previously developed exercise program for knee patients [38], including balance exercises, and resistance exercises (Appendix File 2). The physiotherapist individualized the program according to the patients` impairments (pain, swelling, muscle strength, and neuromuscular control). The dose for the strength exercises was planned according to ACSM`s guidelines for strength progression in healthy adults [37] and an intensity of 8–12 repetitions maximum (RM). The program consisted of a variety of exercises for balance (neuromuscular control) and six exercises for muscle strength for the following muscle groups: quadriceps, hamstring, hip abductors and extensors, and calf muscles. Prior to each exercise session, a warm-up on a stationary bike or a treadmill was performed for 5 min. Each patient’s neuromuscular function was decisive for how and when to advance the neuromuscular exercises, for instance, from standing on both legs to one leg or using different surfaces such as foam mats, wobble boards, or bosu balls. For the strength exercises, the patients were encouraged to follow the “2 + principle”, where the weight load was increased on the next exercise session when the patient was able to perform at least two more repetitions than planned on the last set [39].

#### Aerobic exercise program

The aerobic exercise program was conducted on a stationary cycle and based on guidelines for training parameters in people with pain associated with osteoarthritis [40]. The participants were told to cycle 2–3 times per week for 12 weeks, including a warm-up for 10 min, then 30 min on moderate intensity (70–80% HR_max_) which we considered to be moderate loading on the knee joint), and finish with 5 min on low intensity. The participants were told to have a 2-week preparation phase with low intensity and shorter sessions to avoid “too-much-too-soon” complaints.

#### Usual care

Participants randomized to the usual care group were told to live as they usually did but to avoid starting a *new* exercise program involving structured strength exercise or cycling until the 4-month follow-up was completed. The participants in this group could get access to the exercise program after the post-intervention test was conducted, but < 5 participants in the usual care group asked for the programs.

### Sample size

The sample size was calculated to detect a suggested clinically important difference on the KOOS QoL of 10 points [13] (β = 0.2, two-sided *α* = 0.05) with a standard deviation (sd) of 20 points. This estimation gave 63 in each group, in total of 189 participants. We planned for a total of 69 in each group (n = 207), including a drop-out rate of 10%.

### Statistical methods

The statistical analyses were pre-planned prior to participant enrolment and published at clinicaltrials.gov (NCT01682980) before we revealed the group allocation. Descriptive baseline characteristics are presented for each group separately as number (n) and percentage (%), mean and standard deviations, or median and minimum-maximum (min-max) values depending on the type of the variable or its distribution. All outcomes were compared between each intervention group and the usual care group at the 4-month and 1-year follow-ups. To test the hypotheses for the primary outcome at 1 year, intention-to-treat mixed linear model with restricted maximum likelihood (REML) solution was applied using KOOS QoL data as the dependent variable. Because 7 participants withdrew their informed consents, we had to exclude these from our dataset. Consequently, a modified intention-to-treat analysis was conducted. The baseline score for KOOS QoL were included as covariate, the participant’s ID variable as a random effect, and intervention group, time, and group*time as fixed effect factors. The statistical analyses for secondary outcomes were conducted using the same approach as for the primary outcome. No adjustments for multiplicity were applied. Statistical Package for Social Sciences (IBM© SPSS© Statistics version 27) was used for the statistical analyses. Cost-effectiveness analyses will be reported in a separate paper.

#### Ethical considerations

All participants signed an informed consent prior to the baseline assessment. The Regional Ethical Committee in the Health Region South-East in Norway approved the study protocol (REK 10/223), and the Data Inspectorate at Oslo University Hospital approved the study.

## Results

**Fig. 1:**
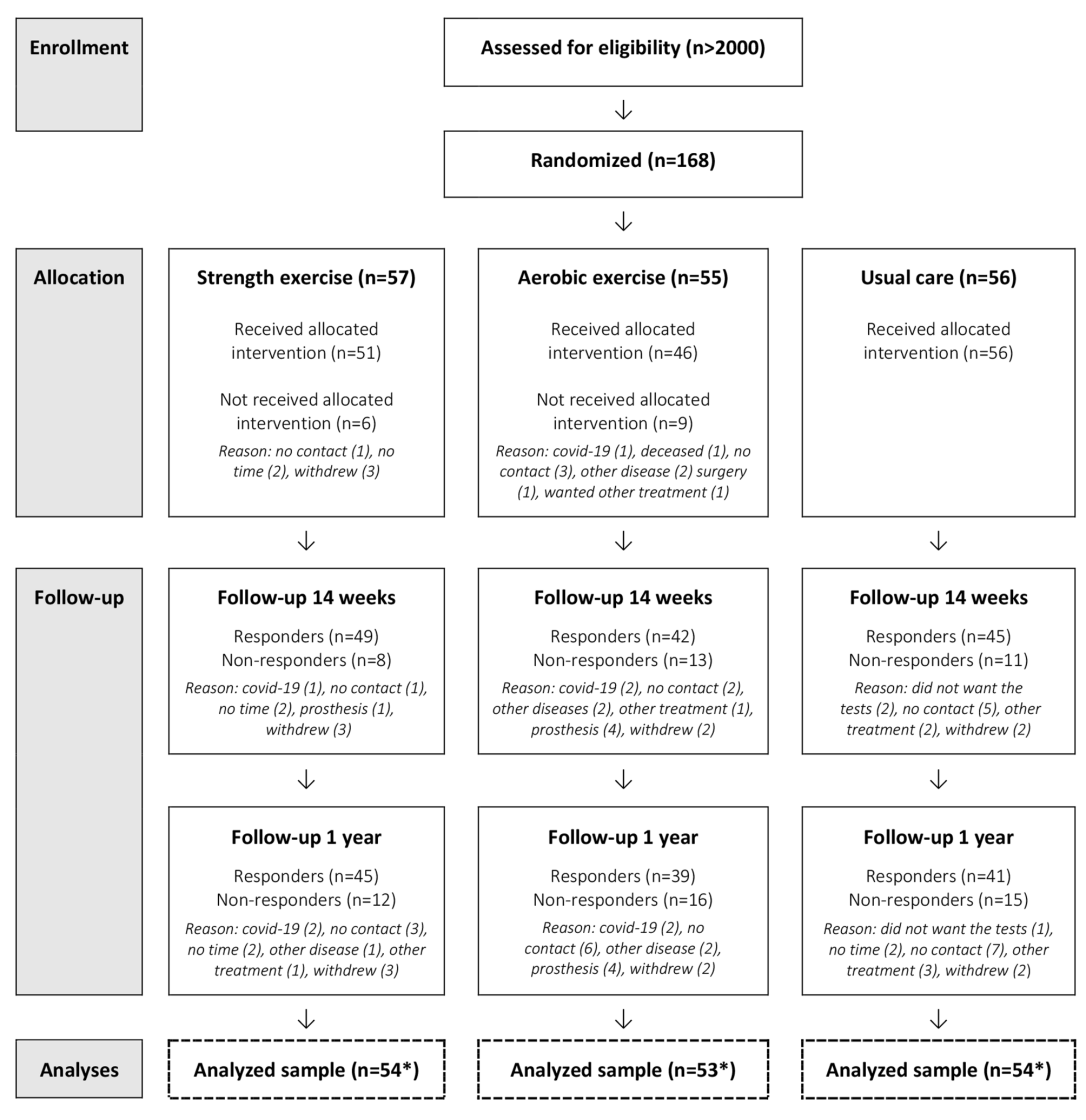
Participant flow during the trial. *Final sample excluded seven participants who withdrew their informed consent to participate (strength exercise: n=3, aerobic exercise: n=2, usual care: n=2)

The trial randomized 168 participants: 55 to strength exercise, 56 to aerobic exercise, and 57 to usual care. Seven participants withdrew their informed consent (due to lack of time) and 19 participants showed up only on the baseline assessment. The participant flow and reasons for loss to follow-up are shown in Fig. 1.

Participants’ baseline characteristics are presented in Table 1. The groups were balanced on baseline characteristics as compared to the usual care group, except for more men and smokers in the usual care group. Sixty-eight physiotherapists treated the participants in the two exercise intervention groups. Training diaries were delivered by 60% in the strength exercise group (33/55) and 57% in the aerobic exercise group (32/56). Of those who delivered diaries, 77% (33 of 43 in the strength exercise group) and 80% (32 of 40 in the aerobic exercise group) completed ≥ 80% of the prescribed training sessions. Physical activity level and most frequent activity type at the three time-points are presented in Appendix File 3. Descriptive data outcomes are given in Table 2.

**Table 1:** Baseline characteristics of the study participants (n = 161)

Characteristics	Strength exercise (n = 54)	Aerobic exercise (n = 53)	Usual care (n = 54)
Sex, men, n (%)	24 (44)	25 (47)	30 (56)
Age (years), mean (sd)	57.6 (6.6)	57.3 (7.1)	57.8 (7.4)
Body mass index, mean (sd)	28.9 (4.3)	29.4 (4.4)	28.4 (4.1)
Smoking, yes, n (%)	2 (4)	6(12)	13 (24)
Education: >4 years of college or university, n (%)	15 (28)	15 (28)	12 (22)
Occupational status, n (%)*			
• Working • Sick leave/Retired	46 (94) 3 (6)	44 (88) 6 (12)	41 (80) 10 (20)
Self-reported knee osteoarthritis*, n (%)			
• One knee • Both knees	31 (60) 21 (40)	39 (75) 13 (25)	32 (60) 21 (40)
Any previous injuries - involved knee, n (%)	26 (48)	30 (57)	16 (30)
Any previous injuries - uninvolved knee	8 (15)	13 (25)	15 (28)
Osteoarthritis in parents or siblings, n (%)	26 (50)	20 (41)	23 (43)
Other pain/injuries (back, hip, ankle), n (%)	11 (20)	16 (30)	10 (19)
Self-reported known heart disease, n (%)	15 (28)	19 (36)	10 (19)
Physiotherapy consultations post-intervention, n (%)			
• 4–6 months • 7–9 months • 10–12 months	11 (22) 9 (18) 5 (12)	15 (33) 11 (26) 9 (24)	12 (27) 17 (38) 14 (37)

N = number, sd = standard deviation, *data had missing values

**Table 2:** Descriptive values for the primary and secondary outcome measures

Outcomes	Strength exercise	Aerobic exercise	Usual Care
**KOOS QoL (0-100)**			
• **Baseline**	**37 (19)**	**33 (15)**	**37 (20)**
• **4 months**	**47 (22)**	**42 (20)**	**39 (23)**
• **1 year**	**47 (25)**	**43 (20)**	**40 (26)**
KOOS Pain (0-100)			
• Baseline	57 (17)	55 (17)	52 (20)
• 4 months	63 (22)	62 (20)	55 (22)
• 1 year	64 (24)	60 (22)	53 (28)
KOOS Symptoms (0-100)			
• Baseline	64 (18)	57 (15)	57 (20)
• 4 months	68 (20)	63 (18)	55 (20)
• 1 year	70 (21)	62 (18)	58 (25)
KOOS ADL (0-100)			
• Baseline	66 (20)	64 (21)	61 (21)
• 4 months	71 (24)	71 (24)	63 (21)
• 1 year	72 (27)	69 (22)	58 (30)
KOOS Sport (0-100)			
• Baseline	31 (22)	27 (19)	27 (24)
• 4 months	42 (27)	33 (22)	30 (26)
• 1 year	37 (27)	33 (32)	33 (32)
Pain last week (0–10)			
• Baseline	4.9 (2.1)	4.6 (1.9)	5.3 (2.3)
• 4 months	3.9 (2.8)	3.7 (2.2)	4.5 (2.4)
• 1 year	4.0 (2.4)	3.7 (2.2)	5.1 (2.9)
EQ-5D-VAS (0-100)			
• Baseline	67 (16)	62 (16)	64 (18)
• 4 months	70 (19)	67 (22)	62 (19)
• 1 year	66 (19)	66 (19)	64 (21)
EQ-5D-5L index			
• Baseline	0.774 (0.159)	0.736 (0.191)	0.725 (0.201)
• 4 months	0.779 (0.180)	0.765 (0.225)	0.747 (0.219)
• 1 year	0.774 (0.194)	0.767 (0.195)	0.696 (0.247)
Self-efficacy for pain (5–25)			
• Baseline	18 (4.6)	17 (4.0)	16 (5.1)
• 4 months	18 (4.8)	18 (4.8)	17 (4.8)
• 1 year	19 (5.2)	18 (5.1)	16 (4.1)
Self-efficacy for symptoms (6–30)			
• Baseline	24 (3.6)	23 (4.1)	22 (4.0)
• 4 months	23 (5.5)	23 (5.1)	22 (5.5)
• 1 year	23 (4.5)	22 (5.5)	20 (5.5)
Quadriceps strength involved knee (Nm)			
• Baseline	122 (51)	116 (40)	108 (42)
• 4 months	133 (53)	129 (42)	108 (46)
• 1 year	125 (46)	129 (36)	112 (48)
Quadriceps strength involved knee (Nm/kg)			
• Baseline	1.4 (0.5)	1.3 (0.3)	1.3 (0.4)
• 4 months	1.6 (0.5)	1.5 (0.3)	1.2 (0.4)
• 1 year	1.6 (0.5)	1.5 (0.3)	1.3 (0.5)
Hamstrings strength involved knee (Nm)			
• Baseline	68 (33)	63 (27)	62 (28)
• 4 months	72 (36)	72 (25)	63 (27)
• 1 year	67 (29)	71 (23)	64 (29)
Hamstrings strength involved (Nm/kg)			
• Baseline	0.8 (0.3)	0.7 (0.3)	0.7 (0.3)
• 4 months	0.8 (0.3)	0.8 (0.2)	0.7 (0.3)
• 1 year	0.7 (0.3)	0.8 (0.2)	0.8 (0.3)
Maximal oxygen consumption (VO_2max_)			
• Baseline	28.2 (7.3)	27.4 (4.9)	29.4 (6.5)
• 4 months	29.1 (7.5)	30.1 (5.9)	28.0 (6.6)
• 1 year	29.6 (8.4)	26.9 (4.1)	27.4 (6.7)

Mean values (standard deviation) are presented. KOOS; Knee injury and osteoarthritis outcomes core, QoL, knee-related quality of life; ADL, activities of daily living; EQ-5D-5 L, EuroQoL-5 Dimensions 5 Levels, VAS, visual analogue scale: 0 best, 10 worst; Nm, newton meter; kg, kilograms (body weight); VO_2max_ was reported in mL*kg*min^− 1^. Numbers at 4 months for KOOS QoL: strength training (n = 49), stationary cycling (n = 42), usual care (n = 45). Numbers At 1 year for KOOS QoL: strength training (n = 45), stationary cycling (n = 39), usual care (n = 41)

### Efficacy of strength exercise or aerobic exercise compared to usual care at 1 year

**Fig. 2:**
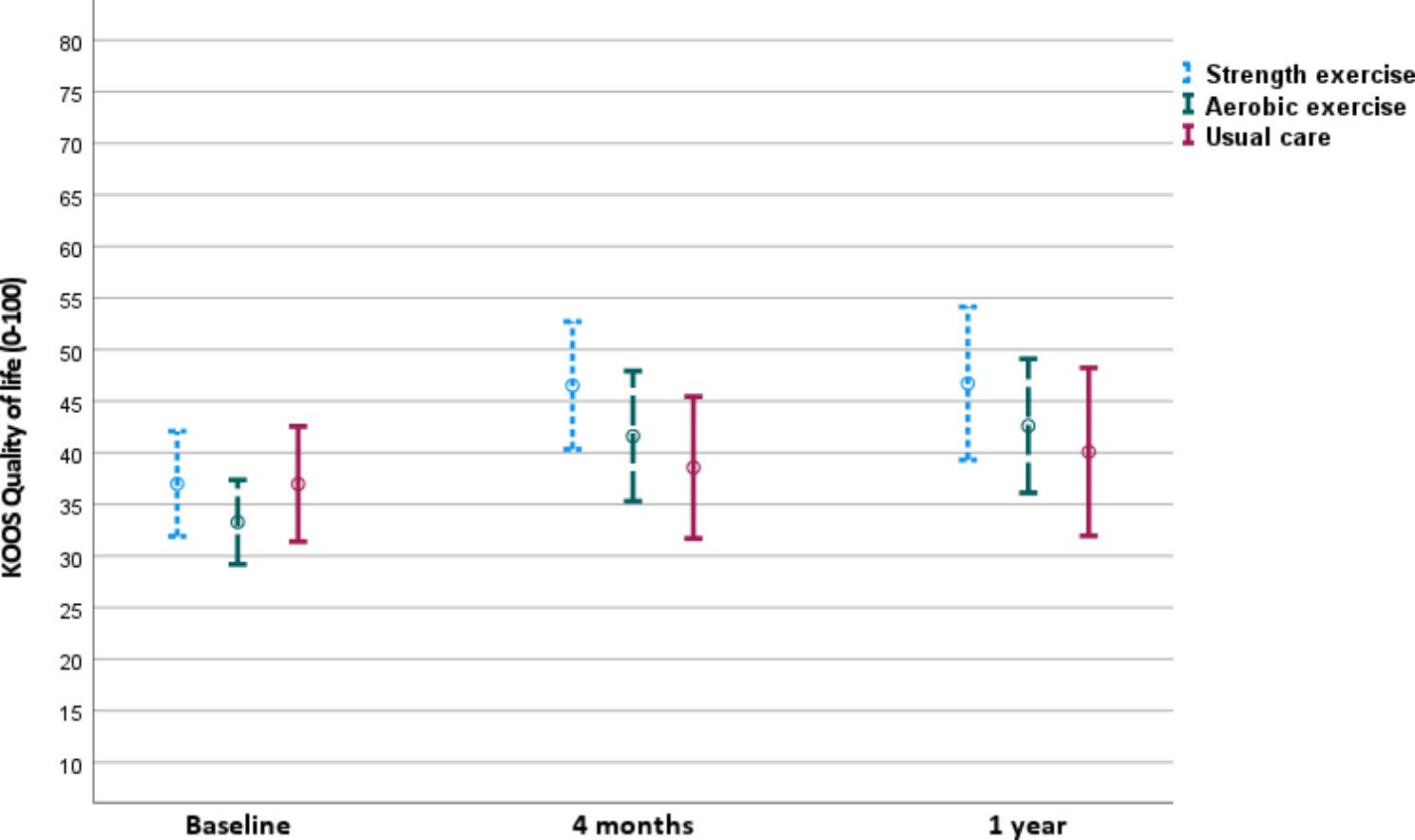
Knee injury and osteoarthritis outcome score (KOOS) knee-related quality of life (QoL) at three time points

There were no statistically significant differences between strength exercise group and usual care group (6.5, 95% CI -0.9 to 14), or between aerobic exercise group and usual care group (5.0, 95% CI -2.7 to 12.8) for KOOS QoL at the 1-year follow-up (Table 3; Fig. 2) beyond the administration of the supervised interventions.

**Table 3:** Mean difference between the intervention groups and the usual care group (UC) (95% CI).

Outcomes	Strength exercise vs. UC	Aerobic exercise vs. UC
**KOOS QoL (0-100)**		
• 4 months	6.7 (-0.5 to 14.0)	4.7 (-2.8 to 12.2)
• 1 year	6.5 (-0.9 to 14.0)	5.0 (-2.7 to 12.8)
KOOS Pain (0-100)		
• 4 months	2.4 (-4.6 to 9.4)	3.2 (-3.9 to 10.4)
• 1 year	5.7 (-1.4 to 12.9)	3.2 (-4.2 to 10.6)
KOOS Symptoms (0-100)		
• 4 months	6.3 (-0.5 to 13.2)	7.4 (0.4 to 14.4)*
• 1 year	6.2 (-0.9 to 13.3)	5.1 (-2.1 to 12.4)
KOOS ADL (0-100)		
• 4 months	2.3 (-4.0 to 9.5)	3.5 (-3.9 to 10.9)
• 1 year	7.3 (-0.1 to 14.6)	5.8 (-1.8 to 13.4)
KOOS Sport (0-100)		
• 4 months	8.4 (-0.1 to 16.9)	2.1 (-6.7 to 10.9)
• 1 year	2.4 (-6.4 to 11.2)	0.5 (-8.6 to 9.5)
Pain last week (0–10)		
• 4 months	-0.4 (-1.2 to 0.4)	-0.3 (-1.1 to 0.6)
• 1 year	-0.9 (-1.7 to -0.02)	-0.9 (-1.8 to 0.03)
EQ-5D-VAS (0-100)		
• 4 months	6.4 (-0.8 to 13.6)	6.4 (-1.1 to 13.9)
• 1 year	2.5 (-5.0 to 9.9)	5.4 (-2.4 to 13.2)
EQ-5D-5L index		
• 4 months	0.00 (-0.07 to 0.07)	0.01 (-0.06 to 0.08)
• 1 year	0.02 (-0.05 to 0.1)	0.06 (-0.02 to 0.13)
Self-efficacy for pain (5–25)		
• 4 months	0.8 (-1.03 to 2.7)	1.0 (-1.0 to 2.9)
• 1 year	2.1 (0.2 to 4.0)*	1.7 (-0.3 to 3.7)
Self-efficacy for symptoms (6–30)		
• 4 months	0.7 (-1.4 to 2.7)	0.6 (-1.5 to 2.7)
• 1 year	2.0 (-0.2 to 4.1)	2.0 (-0.12 to 4.2)
Quadriceps strength (Nm)		
• 4 months	14.3 (5.3 to 23.3)*	10.1 (0.9 to 19.3)*
• 1 year	-1.1 (-11.4 to 7.2)	3.2 (-7.7 to 14.1)
Quadriceps strength (Nm/kg)		
• 4 months	0.21 (0.11 to 0.31)*	0.15 (0.05 to 0.26)*
• 1 year	0.04 (-0.08 to 0.16)	0.04 (-0.08 to 0.16)
Hamstrings strength (Nm)		
• 4 months	3.3 (-2.3 to 9.0)	3.9 (-1.9 to 9.7)
• 1 year	-1.6 (-8.0 to 4.9)	2.4 (-4.4 to 9.3)
Hamstrings strength (Nm/kg)		
• 4 months	0.05 (-0.02 to 0.12)	0.07 (-0.00 to 0.14)
• 1 year	0.00 (-0.08 to 0.9)	0.04 (-0.05 to 0.12)
VO_2max_ (mL·kg^− 1^·min^− 1^)		
• 4 months	1.9 (0.85 to 3.0)*	3.01 (1.9 to 4.1)*
• 1 year	0.85 (-0.5 to 2.2)	0.24 (-1.1 to 1.6)

KOOS; Knee injury and osteoarthritis outcomes core, QoL, knee-related quality of life; ADL, activities of daily living; EQ-5D-5 L, EuroQoL-5 Dimensions 5 Levels, VAS, visual analogue scale: 0 best, 10 worst; Nm, newton meter for involved knee; kg, kilograms (body weight); VO_2peak_, voluntary maximal oxygen consumption. Numbers at 3 months and 1 year varies for the three groups for the different vary. Mixed linear models are adjusted for baseline value of the outcome. *p < 0.05

### Secondary outcomes for strength exercise group vs. usual care group

At the 4-month follow-up, the strength exercise group had statistically significant better scores than the usual care group on quadriceps strength (14.3, 95% CI 5.3 to 23.3 Nm and 0.21, 95% CI 0.11 to 0.31 Nm/BW), and VO_2max_ (1.9, 95% CI 0.85 to 3.0 mL·kg^− 1^·min^− 1^)(Table 3). At the 1-year follow-up, the strength exercise group had statistically significant better results than usual care group for self-efficacy for pain (2.1, 95% CI 0.2 to 4.0) (Table 3).

### Secondary outcomes for aerobic exercise vs. usual care

The aerobic exercise showed better KOOS symptoms score (7.4, 95% CI 0.4 to 14.4), better quadriceps strength (10.1, 95% CI 0.9 to 19.3 Nm and 0.15, 95% CI 0.05 to 0.26 Nm/BW), and better VO_2max_ (3.0, 95% CI 1.9 to 4.1 mL·kg^− 1^·min^− 1^) at the 4-month follow-up as compared to the usual care group (Table 3).

The GRC showed that 65% (at 4 months) and 39% (at 1 year) in the strength exercise group reported a little bit better, much better, or fully recovered knee complaints. In the aerobic exercise group, the corresponding proportions were 62% (at 4 months) and 42% (at 1 year), and in the usual care group 38% (at 4 months) and 31.5% (at 1 year) (Table 4).

**Table 4:** Global rating of change in knee complaints

Categories	Strength exercise		Aerobic exercise		Usual care	
	4 months (n = 46)	1 year (n = 44)	4 months (n = 42)	1 year (n = 38)	4 months (n = 45)	1 year (n = 38)
Completely recovered	1 (2)	0	1 (2)	0	0	2 (5)
Much better	12 (26)	8 (18)	6 (14.5)	6 (16)	5 (11)	7 (18.5)
A little bit better	17 (37)	9 (21)	19 (45.5)	10 (26)	12 (27)	3 (8)
No change	9 (19.5)	16 (36)	7 (17)	11 (29)	20 (44)	13 (34)
A little bit worse	3 (6.5)	8 (18)	7 (17)	9 (24)	5 (11)	7 (18.5)
Much worse	4 (9)	3 (7)	1 (2)	2 (5)	1 (2.5)	4 (11)
Worse than ever	0	0	1 (2)	0	2 (4.5)	2 (5)

Numbers (%) are presented for change in knee complaints from the previous follow-up, between baseline and 4 months and between 4 months and 1 year

#### Adverse events and trial amendments

There were no adverse events during testing or the exercise programs except one participant who experienced high blood pressure and EKG irregularities during the baseline VO_2max_ cycle test. This was inspected by a cardiologist. The participant withdrew the informed consent. Another participant experienced swelling during the cycling, withdrew, and received total knee replacement.

## Discussion

In this RCT we aimed to investigate the efficacy of strength exercise or aerobic exercise compared to usual care on KOOS QoL at 1 year in individuals with symptomatic knee osteoarthritis. Our secondary outcomes included several functional and physiological outcomes at 4 months and 1 year. We did not detect statistically significant between-group differences for knee-related QoL at 1 year, but our results had wide CIs. A recent systematic review evaluating the effects of exercise for patients with knee osteoarthritis [41] summarized results from four studies using KOOS QoL as an outcome measure. In contrast to our study, they did not include 1-year results, but the short-term post-intervention results detected an improvement in QoL. Our study shows inconclusive results due to the wide CIs, which means that the population estimate could be a clinically important effect, or it could be no effect. The same were seen for most of the secondary outcomes. However, the secondary outcome analyses showed that the strength exercise group and the aerobic exercise group had statistically significant better results at the 4-month follow-up for quadriceps strength and VO_2max_. This indicate that the participants responded physiologically to the exercise programs.

Our results are most likely influenced by the smaller sample size than what we intended to include, but the wide CIs might also reflect that the participants have conducted and responded individually to the interventions. Future studies should investigate more thoroughly who is responding to exercise interventions and probable explanations behind clinically important improvements or differences. Furthermore, the lack of efficacy at 1 year might relate to adherence to the exercises both in the intervention period, but also in the post-intervention period. The interventions might not have been optimal for a heterogenous group of patients with knee osteoarthritis with some having previous injury, some were overweight, and they had different experience with moderate intensity training. The physiotherapists were told to tailor the intervention within the frame of the program, but we have no information on how this was conducted other than information from the training diary. We did not collect data on information the physiotherapists gave to the participants during and at the end of the intervention, but we assessed health care utilization at 6 months, 9 months, and 12 months (physiotherapy consultations presented in Table 1). A systematic review with meta-analysis including data from 77 RCTs of hip and knee osteoarthritis patients confirmed exercise benefits for pain, function, QoL, and performance tests at 8 weeks, but with a gradually decreasing effect over time to no better than usual care at around 9 months [9]. The meta-analysis included all types of exercise which preclude direct comparison to our study with strength or cycling interventions. Our exercise groups showed around 10% better quadriceps muscle strength and VO_2max_ at the 4-month follow-up compared to usual care, but no further improvement was seen at the 1-year follow-up. This could indicate that the participants did not continue the structured quadriceps strength exercises and aerobic exercise beyond the intervention period.

### Limitations and strengths

The main limitation of this study is that we did not conduct a feasibility and pilot study before we initiated the trial, thus we had not estimated recruitment rate beforehand. The main reasons for the slow recruitment rate were lack of time to recruit in the primary health care and that only one person recruited participants. Multi-center studies and adequate resources used on the recruitment process should be emphasized to successfully conduct similar RCTs. We decided to terminate the trial early and chose to do that when the COVID-19 pandemic hit Norway in March 2020. The estimates for our outcomes had wide CIs, and with a larger data set, the results may have been different. The clinically important difference for the KOOS QoL with 10 points might not be correct as it has been suggested to be 8 points for “somewhat better” and 15.6 points for a great deal better [42], but these limits are not well established. Furthermore, KOOS QoL might be a challenging outcome for measuring efficacy after exercises (for instance by influencing pain and function more directly than QoL), even though it has been more sensitive for changes in this population. The KOOS QoL was used as primary outcome because this score has been found to have a greater responsiveness in younger populations compared to other instruments such as WOMAC and the SF-36 [13]. We have no data on potential eligible patients seeking health care for knee osteoarthritis in the institutions and hospitals over the recruitment period. We have not recorded reasons for ineligibility because we received patients from clinical practice and advertisements. The physical activity level data indicated that the participants were active before the intervention. However, their main activity was walking, and participants who reported structured strength training or cycling regularly were not included. Our experiences from the recruitment conversations were that the participants generally overreported their physical activity level, particularly the intensity. Low physical activity was also confirmed by the fact that most participants expressed that the cycling VO_2max_ test was unexpectable heavy. We excluded participants with a BMI > 35 because we believe an intervention for that group needs to include a weight reduction intervention in addition to exercise. We also excluded eligible participants above 70 years because we aimed to target a younger osteoarthritis population. The exercise interventions lasted around 90 to 180 min per week, which may have been too little to improve KOOS QoL and knee function; however, to the best of our knowledge, at the time when we designed the study, no studies had documented the best dosage beyond the recommendations given by WHO guidelines of 150 min of exercise per week. Juhl et al. reported in 2014 [6] that an optimal exercise program for improving pain and disability in knee osteoarthritis should be supervised and conducted three times per week. However, the review did not include KOOS QoL.

The strength of this study is that we have included relatively young study participants from primary and secondary care and conducted the study in clinical practice. We analyzed data with a mixed-effect linear model that includes participants regardless of one missing follow-up, and includes both fixed and random factors. This study was designed at a time point when user involvement was not a mandatory part of trials, however, the strength exercise program was developed collaboration with specialists in sport physical therapy with high competence in treating patients with knee osteoarthritis. Furthermore, according to the systematic review by Luan et al. [15] few studies have evaluated the effect of aerobic exercise, such as cycling exercise on QoL in patients with knee osteoarthritis. To our knowledge, none have tested direct maximal oxygen consumption.

### Clinical implications

This study showed no effect of exercise compared to usual care on knee-related QoL at 1 year. Secondary outcomes showed that strength and aerobic exercise programs improved physiological measures at 4 months, but not at 1 year. Importantly, cycling 2–3 times a week at moderate intensity showed a 10% improved VO_2max_ which could be important for general health and lifestyle diseases in this population. Longer follow-up with supervision from physiotherapists might be needed to maintain the short-term, post-intervention physiological effects. Future studies might be more personalized adding behavioral change and self-management strategies to people who struggle with exercise adherence, and future studies should investigate which patients respond to exercise interventions.

## Conclusion

This trial found no statistically significant effects of two exercise programs compared to usual care on KOOS QoL at 1 year in individuals with knee osteoarthritis. An underpowered sample size may explain lack of efficacy between the intervention and the usual care groups and imprecise results. Secondary outcomes showed improved quadriceps strength and VO_2max_ at 4 months in both groups.

### Electronic supplementary material

Below is the link to the electronic supplementary material.

Appendix 1

Appendix 2

Appendix 3

## Data Availability

The dataset for replication analyses of this paper may be available in an anonymous format according to GDPR. Please contact the corresponding author for requests.
