# Supplementary material for: The efficacy of strength or aerobic exercise on quality of life and knee function in patients with knee osteoarthritis. A multi-arm randomized controlled trial with 1-year follow-up
Source: BMC Musculoskelet Disord. 2023 Sep 8;24:714. doi: 10.1186/s12891-023-06831-x (PMC10485991; doi:10.1186/s12891-023-06831-x)
Supplement: Supplementary file 1 — Appendix 1 [file 12891_2023_6831_MOESM1_ESM.pdf]

CERT ✓ Consensus on Exercise Reporting Template

A Checklist for what to include when reporting exercise programs

Section/Topic	Item #	Checklist item	Location ** Primary paper (page, table, appendix)	† Other (paper or protocol, website (URL)
**WHAT: materials**	1	Detailed description of the type of exercise equipment (e.g. weights, exercise equipment such as machines, treadmill, bicycle ergometer etc)	10-12	Appendix III
**WHO: provider**	2	Detailed description of the qualifications, teaching/supervising expertise, and/or training undertaken by the exercise instructor	9	
**HOW: delivery**	3	Describe whether exercises are performed individually or in a group	11-12	
	4	Describe whether exercises are supervised or unsupervised and how they are delivered	10-11	
	5	Detailed description of how adherence to exercise is measured and reported	11	
	6	Detailed description of motivation strategies	n.a	
	7a	Detailed description of the decision rule(s) for determining exercise progression	11	
	7b	Detailed description of how the exercise program was progressed		Appendix III
	8	Detailed description of each exercise to enable replication (e.g. photographs, illustrations , video etc)		Appendix III
	9	Detailed description of any home program component (e.g. other exercises, stretching etc)		Appendix III
	10	Describe whether there are any non-exercise components (e.g. education, cognitive behavioural therapy, massage etc)	10	
	11	Describe the type and number of adverse events that occurred during exercise	16	

**WHERE: location**	12	Describe the setting in which the exercises are performed	10–11	
**WHEN, HOW MUCH: dosage**	13	Detailed description of the exercise ~~intervention including, but not~~ limited to, number of exercise repetitions/sets/sessions, ~~session duration, intervention~~/program duration etc	10–11	Appendix III
**TAILORING: what, how**	14a	Describe whether the exercises are generic (one size fits all) or tailored whether tailored to the individual	11–12	
	14b	Detailed description of how exercises are tailored to the individual		
	15	Describe the decision rule for determining the starting level at which people commence an exercise program (such as beginner, intermediate, advanced etc)		
**HOW WELL: planned, actual**	16a	Describe how adherence or fidelity to the exercise intervention is assessed/measured		
	16b	Describe the extent to which the intervention was delivered as planned		

***It is recommended that this checklist is used in conjunction with the Explanation and Elaboration Statement which is a guide each item in the CERT Checklist**

The CERT Checklist is designed for reporting details of an exercise intervention. The CERT Checklist should be used in conjunction with a reporting checklist appropriate for the study type e.g. the CONSORT Statement (www.consort-statement.org) for randomised controlled trials, the SPIRIT Statement (www.spirit-statement.org) for a clinical trial protocol. For further guidance regarding reporting guidelines please consult the EQUATOR network (www.equator-network.org)

** Authors – please use N/A if an item is not applicable Reviewers – please use ”?” if information is not provided or not/insufficiently reported

† If the information is not provided in the primary paper that is under consideration, please provide details of where this information is available e.g. in a published protocol, published papers (provide citation details) or on a website (provide the URL).
