# Supplementary material for: The efficacy of strength or aerobic exercise on quality of life and knee function in patients with knee osteoarthritis. A multi-arm randomized controlled trial with 1-year follow-up
Source: BMC Musculoskelet Disord. 2023 Sep 8;24:714. doi: 10.1186/s12891-023-06831-x (PMC10485991; doi:10.1186/s12891-023-06831-x)
Supplement: Supplementary file 2 — Appendix 2 [file 12891_2023_6831_MOESM2_ESM.docx]

| **WARM UP** | |  |  |
| --- | --- | --- | --- |
| **Treadmill**  **** | Walk or jog on a treadmill for 5 minutes. Adjust the treadmill to 2% of inclination.  Stationary bike is an alternative |  |  |
| **NEUROMUSCULAR EXERCISES** | |  | **PROGRESSION** |
| **Standing balance exercises** | Stand up straight with feet shoulder width apart. Put weight on the right foot, and then transfer your body weight to the left foot. Repeat. |   |      Progression: use a wobble cushion or a balance mat |
| **Step-up exercises** | Standing in front of a step or stair-case: Place one foot onto the step. Transfer bodyweight to the front foot, lean forward and push slowly up with all bodyweight on front leg. Go slowly back down. Repeat 3 x 10 on both legs. |   |  |
| **Single leg exercises** | Standing on one leg, lift the other leg up until hip and knee are in 90 degrees, and slowly down to the start position and repeat. |   |    Progression: do the exercise on a balance cushion or use a resistance band. |
| **STRENGTH EXERCISES** |  |  | **PROGRESSION** |
| **Squat exercises**    | 3 x 8 repetitions (reps).  Standing with shoulder-width apart. Squat down, keep your back straight, hips back and torso forward. The weight should be on the heels, not the toes. |   | Include a barbell across the back of your shoulders. Increase the load when you manage more than 3 x 8 reps.  Squats with ball against a wall: squat slowly down and up. When you manage to do more than 3x15 reps, include weights in your hands |
| **Leg extension exercises**   | 3 x 8 reps. |   | Increase the load when you manage to do more than 3 x 8 reps. |
| **Bridge with pelvic tilt** **exercises**   | 3 x 10 reps. Supine position. Feet and arms on the floor. Knees bent and feet flat on the floor. Tilt the pelvis and lift the hips up while still holding the pelvis level. Hold the position before returning slowly to the floor. |     | Lift one leg up. Keep the pelvis level and hold 5 seconds before returning the leg to the floor.  Do the same exercise with both legs on a Bosu ball. |
| **Lunge exercises**  ** ** | Step forward with the exercising leg, keeping trunk upright with a position of 90° hip and knee flexion. Return to the starting position. Repeat on the other leg |    | Forward lunges with weights down by the side. Press back up. Repeat on the other leg.  Split squat rear foot elevated: Create a large distance between the front and the back foot. Keep the back straight. Bend the knee and drop the hips straight down towards the ground. Push back up. Repeat on the other leg. Use weights to progress. |
| **Hip abduction exercises**  **** | 3 x 15 reps.  Side lying, a straight line from the head through the trunk, down the legs to the toes. Keep your arm under the head. Lift the top leg straight up. |   | Side lying, hip abduction exercise with weights. Do 3 x 10 reps and increase the load when you are able to do more than 3 x 10 reps. |
| **Standing heel raise exercises**    | In a machine: 3 x 8 reps  Without a machine: 3 x 15 reps |    | In a machine: Increase the load when you can do more than 3 x 8 reps.  Without a machine: Increase the load by carrying something in your hands or in a back-pack, or do single leg heel raise. |

| **HOME EXERCISES** |  |  | **PROGRESSION** |
| --- | --- | --- | --- |
| **Warm up** | Walk, jog or ride a stationary bike for 5 minutes. |  |  |
| **Bridge with pelvic tilt** **exercises**   | 3 x 10 reps. Supine position. Feet and arms on the floor. Knees bent and feet flat on the floor. Tilt the pelvis and lift the hips up while still holding the pelvis level. Hold the position before returning slowly to the floor. |   | Lift one leg up. Keep the pelvis level and hold 5 seconds before returning the leg to the floor.  Do the same exercise with both legs on a Bosu ball. |
| **Hip sbduction exercises**   | 3 x 15 reps.  Side lying, a straight line from the head through the trunk, down the legs to the toes. Keep your arm under the head. Lift the top leg straight up. |   | Side lying, hip abduction exercise with weights. Do 3 x 10 reps and increase the load when you are able to do more than 3 x 10 reps. |
| **Squat exercises**    | 3 x 8 reps.  Standing with shoulder-width apart. Squat down, keep your back straight, hips back and torso forward. The weight should be on the heels, not the toes. |   | Include a barbell across the back of your shoulders. Increase the load when you manage more than 3 x 8 reps.  Squats with ball against a wall: squat slowly down and up. When you manage to do more than 3x15 reps, include weights in your hands |
