# Supplementary material for: The efficacy of strength or aerobic exercise on quality of life and knee function in patients with knee osteoarthritis. A multi-arm randomized controlled trial with 1-year follow-up
Source: BMC Musculoskelet Disord. 2023 Sep 8;24:714. doi: 10.1186/s12891-023-06831-x (PMC10485991; doi:10.1186/s12891-023-06831-x)
Supplement: Supplementary file 3 — Appendix 3 [file 12891_2023_6831_MOESM3_ESM.docx]

**Appendix File 3. Physical activity level**

| **Characteristics** | **Strength training** | **Cycling** | **Usual care** |
| --- | --- | --- | --- |
| **BASELINE** |  |  |  |
| Sessions per week*, n (%)   - None - <1 session per week - One session per week - 2-3 sessions per week - Daily sessions | 4 (7)  8 (15)  10 (19)  27 (50)  5 (9) | 1 (2)  11 (21)  11 (21)  17 (32)  13 (25) | 5 (9)  6 (11)  15 (28)  22 (41)  6 (11) |
| Most frequent activity type**   - Walking - Cycling - Strength training - Other (skiing, golf, aerobic, soccer) | 26 (54)  11 (23)  4 (8)  7 (15) | 31 (61)  13 (25)  5 (10)  2 (4) | 26 (53)  16 (33)  3 (6)  4 (8) |
| Intensity per session*   - Easy - Hard (breathing and sweating) - Extremely hard (until exhaustion) | 14 (29)  33 (69)  1 (2) | 26 (50)  25 (48)  1 (2) | 25 (50)  25 (50)  0 |
| Duration per session**   - Less than 15 minutes - 16-30 minutes - 30 minutes to 1 hour - More than 1 hour | 2 (4)  9 (19)  29 (60)  8 (17) | 1 (2)  9 (17)  35 (67)  7 (14) | 1 (3)  14 (28)  27 (54)  8 (16) |
| Physical activity level index (0-15), median (min-max) | 1.9 (0-7.5) | 3.8 (0-10) | 1.9 (0-7.5) |
| **4 months** |  |  |  |
| Sessions per week*, n (%)   - None - <1 session per week - One session per week - 2-3 sessions per week - Daily sessions - Missing | 1 (2)  3 (6)  2 (4)  36 (74)  7 (14)  5 | 0  4 (9)  6 (14)  21 (50)  11 (26)  11 | 1 (2)  6 (13)  10 (22)  21 (47)  7 (16)  9 |
| Most frequent activity type**   - Walking - Cycling - Strength training - Other (skiing, golf, aerobic, soccer) - Missing | 15 (28)  11 (20)  15 (28)  6 (14)  7 | 17 (40)  19 (44)  4 (9)  3 (7)  10 | 20 (47)  13 (30)  7 (16)  3 (7)  11 |
| Intensity per session*   - Easy - Hard (breathing and sweating) - Extremely hard (until exhaustion) - Missing | 13 (28)  32 (68)  2 (4)  7 | 11 (26)  28 (67)  3 (7)  11 | 18 (42)  25 (58)  0  11 |
| Duration per session**   - Less than 15 minutes - 15-30 minutes - 30 minutes to 1 hour - More than 1 hour - Missing | 0  4 (8)  34 (72)  9 (19)  7 | 0  10 (19)  24 (57)  8 (19)  11 | 0  12 (28)  23 (54)  8 (19)  11 |
| Physical activity level index (0-15), median (min-max) | 3.75 (0-10) | 3.75 (0.4-11.2) | 1.89 (0-7.5) |
| **1 year** |  |  |  |
| Sessions per week*, n (%)   - None - <1 session per week - One session per week - 2-3 sessions per week - Daily sessions - Missing | 0  4 (9)  7 (16)  25 (57)  8 (18)  10 | 1 (3)  3 (8)  10 (26)  16 (41)  9 (23)  14 | 0  4 (10)  11 (27)  19 (46)  7 (17)  13 |
| Most frequent activity type**   - Walking - Cycling - Strength training - Other (skiing, golf, aerobic, soccer) - Missing | 20 (47)  7 (16)  13 (30)  3 (7)  11 | 15 (41)  13 (35)  7 (19)  2 (5)  16 | 17 (42)  11 (28)  5 (12)  7 (18)  14 |
| Intensity per session*   - Easy - Hard (breathing and sweating) - Extremely hard (until exhaustion) - Missing | 14 (33)  29 (67)  0  11 | 6 (17)  27 (75)  3 (8)  17 | 23 (57)  16 (40)  1 (3)  14 |
| Duration per session**   - Less than 15 minutes - 16-30 minutes - 30 minutes to 1 hour - More than 1 hour - Missing | 3 (7)  9 (21)  25 (57)  7 (16)  10 | 0  8 (21)  22 (58)  8 (21)  15 | 2 (5)  7 (18)  23 (58)  8 (20)  14 |
| Physical activity level index (0-15), median (min-max) | 2.5 (0.1-10) | 3.75 (0-11.3) | 1.89 (0.1-5.0) |

*data had missing values, **some participants had no sessions per week. Physical activity level index were calculated based on sessions per week, intensity and duration using a formula reported by Kurtze et al. 2008: Reliability and validity of self-reported physical activity in the Nord-Trøndelag Health Study –HUNT 1. Scand J Public Health 2008 36:52. DOI 10.1177/1403494807085373.
